# The therapeutic potential of *Astragalus membranaceus* in atopic dermatitis: from traditional applications and modern pharmacological research to regulation of the Gut-Skin Axis

**DOI:** 10.3389/fphar.2025.1685708

**Published:** 2025-11-07

**Authors:** Chengshuang Lu, Yuping Zeng, Guo Wang, Buqing Lou, Yifan Wang, Wancheng Liu, Zhiming Yan, Haoyang Fu

**Affiliations:** 1 Second Clinical Medical College, Guangzhou University of Chinese Medicine, Guangdong, China; 2 Guangdong Provincial Hospital of Traditional Chinese Medicine, Guangdong, China; 3 Guangdong Pharmaceutical University, Guangdong, China; 4 Provincial-Ministry Jointly-Built National Key Laboratory for Traditional Chinese Medicine Wet Syndrome, Guangdong, China

**Keywords:** *Astragalus membranaceus*, atopic dermatitis, Gut-Skin Axis, immunity, inflammation, computer simulation

## Abstract

Atopic dermatitis (AD) is a difficult-to-treat and recurrent skin condition that often imposes a heavy burden on patients and healthcare systems due to the high costs associated with its treatment and management. *Astragalus membranaceus* (AM), as a botanical drug, has been shown to alleviate skin diseases through multiple mechanisms. However, its systematic mechanism of action against AD remains unclear. This research summarizes the molecular mechanisms through which AM and its active components (polysaccharides, saponins, flavonoids) mitigate AD. The study proposes, for the first time, that AM may alleviate the onset and progression of AD by inhibiting the translocation of gut-derived inflammatory factors to the skin through the Gut-Skin Axis (GSA). Through comprehensive analysis of network pharmacology, molecular docking, and molecular dynamics simulations, compounds with potentially high activity of AM were preliminarily screened. The potential interaction mechanism between this compound molecule and the target protein in AD treatment was further explored. A total of 89 common targets were identified between AM and AD. Enrichment analysis suggests that signaling pathways such as IL-6, TNF-α, NF-κB, and IL-17 may serve as key regulatory hubs in the progression of AD. At conventional doses, AM exhibits a good safety profile. However, the risk of interactions when combined with traditional AD treatments (such as tacrolimus) warrants attention, necessitating enhanced safety evaluations before clinical application. Overall, AM holds potential as an adjunctive therapy for mitigating side effects and improving symptoms, offering a safer alternative to existing treatments. It contributes to shifting AD treatment strategies from purely symptom control toward addressing both symptoms and underlying causes.

## 1 Introduction

Atopic dermatitis (AD, atopic eczema) is a chronic inflammatory skin disease of exceptionally high global prevalence, with a prevalence of up to 20% among children in Europe and Asia (Yew et al., 2025). Its main clinical features are recurrent eczematous lesions manifesting as dry skin, infiltrative erythema, papules, exudation, and intense itching (Langan et al., 2020). In addition to skin symptoms, AD patients often suffer from comorbidities such as depression, anxiety, and sleep disorders, which severely affect their quality of life (Ferrucci et al., 2021). AD arises from a complex interplay of multiple factors, including genetic predisposition, impairment of the epidermal barrier, immune system dysregulation, and microbial imbalance (Guttman-Yassky et al., 2025). “Atopy” refers to an inherited susceptibility to allergic disorders, characterized by an exaggerated immune response to common allergens. Clinically, the classic progression of atopic disease typically begins with the onset of AD, which may subsequently be followed by asthma and allergic rhinitis. This disease sequence is termed the allergic March (also known as the atopic triad) (Hill and Spergel, 2018). Mounting evidence from clinical and genetic studies reveals that the risk of developing further atopic disorders is markedly elevated among patients suffering from severe AD relative to the broader population (Paller et al., 2018). Persistent itching and prolonged disease progression impose a heavy physical, psychological, and economic burden on patients. At the same time, as the initial stage of the atopic process, early effective control of AD is crucial for interrupting the allergic process and reducing the risk of systemic comorbidities. The current standard treatment regimen for AD primarily includes topical or systemic immunosuppressants (such as corticosteroids and tacrolimus) and biologics. While these medications effectively control symptoms, long-term use is often accompanied by side effects, including skin atrophy, metabolic abnormalities, immunosuppression, and potential dependency. Furthermore, some patients exhibit poor response to existing therapies, leading to frequent disease recurrence. Current therapies predominantly focus on symptom management, with insufficient intervention targeting the underlying mechanisms of the disease, such as immune microenvironment imbalance and dysregulation of the GSA. This underscores the urgent need for novel therapeutic strategies that combine multi-target regulation with long-term safety.

The botanical drug *Astragalus membranaceus* [Leguminosae, *Astragalus membranaceus* Fisch. ex Bunge] (AM) possesses dual medicinal and edible value. It has the effects of tonifying qi, solidifying the exterior, promoting wound healing and tissue regeneration, and expelling toxins and pus. It is a classic botanical drug used in traditional Chinese medicine (TCM) to treat skin conditions, including boils, abscesses, and ulcers. Modern pharmacological research has confirmed that AM exhibits a range of medicinal activities, including immune regulation, anti-inflammatory effects, antioxidant stress protection, and antitumor properties (Li et al., 2025). AM has a complex and diverse chemical composition, with polysaccharides, saponins, and flavonoids being the main active ingredients (Dong et al., 2023; Zhang and Tian, 2022). Astragalus polysaccharides (APS), the predominant chemical constituent in AM, primarily comprises glucans and heteropolysaccharides (Liu X. Y. et al., 2024). It has a variety of biological activities, such as immune modulation, blood glucose regulation, antitumor, anticancer, and antiaging, among which immune modulation is the most prominent (Li et al., 2022). Astragaloside IV (AS-IV) is an important monomeric component of AM. It has been established as a key indicator for assessing the quality of AM medicinal botanical drugs in the *Chinese Pharmacopoeia* and is considered to be the core substance responsible for the pharmacological effects of AM. Flavonoids mainly include flavonols (e.g., kaempferol, quercetin) and isoflavonoids (e.g., calycosin, formononetin) (Yu et al., 2018). Although numerous studies have revealed the therapeutic potential and mechanisms of AM active components for treating AD, these findings remain fragmented. The integrated mechanisms involving multiple components, targets, and pathways—particularly the holistic regulatory network based on GSA—have yet to be systematically elucidated.

Given the complex composition and highly systemic mechanisms of action of AM, traditional experimental methods struggle to comprehensively reveal its holistic regulatory network. Computational biology approaches such as network pharmacology, molecular docking, and molecular dynamics simulations can efficiently integrate component-target-pathway data to predict the multi-target synergistic mechanisms of TCM at the systems level. Therefore, building upon a synthesis of AM’s traditional applications and modern pharmacological evidence, this study pioneers the integration of computational simulation methods to systematically elucidate its multidimensional intervention mechanisms in AD. These mechanisms encompass immune modulation, anti-inflammation, barrier repair, and GSA regulation, aiming to provide directional guidance and novel perspectives for in-depth research and clinical translation of AM in AD treatment.

## 2 Pathophysiological mechanisms of AD

Among the known risk factors for AD, family history poses a considerable risk. If parents have asthma, allergic rhinitis, or food allergies, a 1.5-fold increase in the risk of developing AD is observed in their offspring. In cases where a father or mother has AD, the risk increases by 2–3 times, and if both parents have AD, the risk increases by 3–5 times (Wienholtz et al., 2025). Filaggrin (FLG) mutations drive susceptibility to AD through impairment of the skin barrier, promoting the penetration of external allergens, and releasing thymic stromal lymphopoietin (TSLP) (Furue et al., 2017; Meledathu et al., 2025). The disruption of skin barrier function serves as the initiating factor for the core pathology of AD. This defect not only directly drives degradation of intercellular junctions, increased transepidermal water loss (TEWL), and elevated skin surface pH (Tsakok et al., 2019) but also triggers a cascade of key pathological processes, including dysbiosis, inflammatory cell infiltration, epidermal thickening, and upregulation of Th2 cytokines (Das et al., 2022; Kim and Leung, 2018; Li et al., 2021). Continuous pruritus is a hallmark symptom of AD. The mechanical injury from scratching triggers keratinocyte-derived inflammatory signaling, activating Th2-mediated immune responses and stimulating pruritogen release. The binding of these pruritogens to pruriceptive neurons establishes a self-perpetuating “itch-scratch-itch” cycle (Yosipovitch et al., 2020; Yosipovitch et al., 2018). In the long-term course of AD, repeated inflammatory stimulation and mechanical scratching work together to cause further characteristic tissue remodeling phenomena such as epidermal thickening and lichenification (Criado et al., 2024; Sroka-Tomaszewska and Trzeciak, 2021). Keratinocytes, as key regulators of epidermal homeostasis, can disrupt the epidermal barrier when their function is abnormal and can mediate immune microenvironment imbalance through the release of cytokines (Hatano and Elias, 2023). Activated keratinocytes produce pro-inflammatory cytokines (such as TSLP, IL-1β, IL-25, and IL-33) and chemokines, establishing an inflammatory microenvironment and transmitting pruritus signals (Asahina and Maeda, 2017; Das et al., 2022). TSLP, as an epidermal-derived cytokine, plays a crucial role in the initiation and maintenance of inflammation in AD by activating dendritic cells and promoting Th2-type immune responses (Sikorska-Szaflik et al., 2025). IL-33 and IL-25 can further activate type 2 innate lymphoid cells (ILC2s), which in turn produce IL-5 and IL-13, activating eosinophils and Th2 cells (Ständer, 2021). Within the AD skin inflammatory microenvironment, infiltrating basophils serve as a key source of peripheral IL-4, whereas IL-5 acts as the most potent mediator for eosinophil function (Haddad et al., 2022; Roufosse, 2018).

The course of AD shows a phased cytokine shift: Th2 polarization is obvious in the acute phase, while Th1, Th17, and Th22 pathways are synergistically enhanced in the chronic phase (Kim, 2022). Differentiated Th2 cells express Th2-derived cytokines, such as IL-4, IL-13, and IL-31, which induce B cells to produce Immunoglobulin E (IgE) (Ständer, 2021). IL-31 is a cytokine released by Th2 cells and is associated with itching in AD. IL-31 triggers the “itch-scratch” cycle by mediating neuroimmune interactions and promotes epidermal hyperplasia and thickening, exacerbating barrier dysfunction (Fassett et al., 2023; Singh et al., 2016). Th1 cells primarily secrete cytokines such as interferon (IFN)-γ and tumor necrosis factor (TNF)-α to mediate the host’s immune response (Furue et al., 2017; Sroka-Tomaszewska and Trzeciak, 2021). Th17 and Th22-mediated immune responses are often involved in the pathogenesis of inflammatory skin diseases such as AD (Lee et al., 2019). Elevated levels of IL-17, a cytokine produced by Th17 cells, are found in the peripheral blood of AD patients and correlate with more severe disease (Sugaya, 2020). IL-17 inhibits the synthesis of FLG in keratinocytes, damaging skin integrity and barrier function (Liu et al., 2020). Regulatory T (Treg) cells are key regulators of immune tolerance and homeostasis. Dysfunction of these cells can lead to various immune disorders, including autoimmune diseases and allergies. Modulating the number or function of Treg cells offers therapeutic potential for treating autoimmune diseases.In AD, a distinct Th17/Treg immune imbalance exists. This imbalance correlates with disease severity and serum IgE levels, potentially playing a significant role in the pathogenesis of AD. IL-22 levels are significantly elevated in AD patients and are a key factor in driving the inflammatory response, disrupting skin barrier function, and ultimately leading to the characteristic symptoms of AD (Laska et al., 2024). The colonization rate of *S. aureus* (*S. aureus*) is significantly higher on the skin of patients with AD, correlating with disease severity and showing evidence of intra-family transmission (Gu, 2023). *S. aureus* originating from AD lesions disrupts skin barrier function through microbial imbalance, colonization advantage, and release of toxins such as δ-toxin and superantigens (Geoghegan et al., 2018). It can not only directly invade epidermal keratinocytes but also induce Th2-type immune skewing through antigen presentation by Langerhans cells, interfering with the Th1, Th2 lymphocyte polarization balance (Iwamoto et al., 2019; Kim et al., 2019).

**FIGURE 1:**
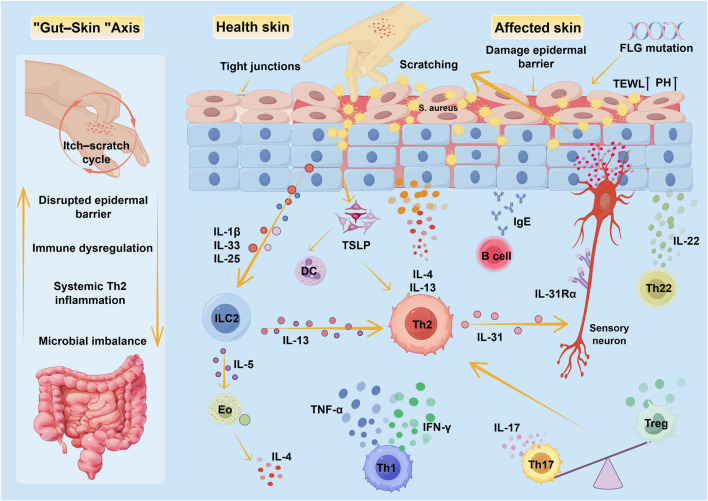
Pathogenesis of AD.

As research into the GSA deepens, the close connection between gut microbiota and skin health has become increasingly clear, and its significant role in the onset and progression of AD has gradually been revealed. There is growing evidence that gut flora disorders, along with their mediated intestinal barrier dysfunction (leaky gut) and systemic inflammation, can profoundly affect skin immune homeostasis and barrier function through the GSA pathway (Chun et al., 2021). Gut microbial diversity and the abundance of butyrate-producing bacteria show a significant inverse relationship with the severity of AD, also further providing key evidence for the pathogenic mechanisms of the GSA (Nylund et al., 2015). Therefore, the migration of gut-derived systemic inflammation to the skin via this pathway is considered an important factor in driving AD chronicity and exacerbating its severity. Figure 1 Pathogenesis of AD.

Figure 1 summarizes the core mechanisms underlying AD pathogenesis. The pathological process of AD may originate from genetic or acquired epidermal barrier defects, manifested as disrupted tight junctions between keratinocytes, increased transepidermal water loss, and pH imbalance. These alterations promote the establishment of specific colonization dominance by *Staphylococcus aureus* and *Staphylococcus* epidermidis. Abnormally activated keratinocytes release inflammatory mediators such as TSLP and IL-33, activating a Th2 immune response that drives an IL-4/IL-13-dominant Th2 inflammatory cascade. Concurrently, IL-31 exacerbates skin barrier damage by activating pruritus-related neural signaling circuits (the itch-scratch cycle). During the chronic phase, Th1-derived IFN-γ/TNF-α drives delayed-type hypersensitivity reactions, while Th17-derived IL-17 and Th22-produced IL-22, respectively, exacerbate dysbiosis and promote epidermal hyperkeratinization. Gut microbiota additionally, a marked Th17/Treg immune imbalance was observed throughout the immune response process, dysbiosis disrupts the intestinal barrier, leading to the translocation of microbial metabolites and endotoxins. Through the GSA, this activates systemic Th2 polarization, further exacerbating skin inflammation and barrier damage.

Eo: eosinophil, IFN-γ: interferon-γ, IL: interleukin, ILC: Innate lymphoid cell, Th: T-helper cell, FLG: Filaggrin, DC: dendritic cell.

## 3 TCM treatment of AD

### 3.1 TCM etiology and pathogenesis of AD

TCM has significant advantages in alleviating eczema symptoms and regulating immune function. It can be used to intervene in the development of atopic processes, improve existing clinical manifestations, and effectively reduce disease recurrence rates and hormone dependence (Liu D. et al., 2025; Wang et al., 2021). Although TCM practitioners have different views on the etiology and pathogenesis of AD, most agree that its root cause lies in a congenitally weak constitution that leads to poor spleen digestion, causing damp-heat to accumulate within the body, which ultimately affects skin health. Therefore, TCM often emphasizes the importance of strengthening the spleen in treating AD (Chen, 2013). Additionally, for patients with chronic or recurrent AD, prolonged disease progression depletes yin fluids and blood, or an inherent yin deficiency may lead to blood deficiency generating wind and transforming into dryness that injures yin, resulting in the pattern of “blood deficiency with wind-dryness.” (Zhu et al., 2021). Clinically, patients primarily present with dry skin, scaling, intense itching, and lichenification.

The concept of “the lung and large intestine are interiorly-exteriorly related” in TCM also provides a crucial perspective for understanding the systemic pathogenesis of AD. The lung governs the skin and hair, regulating the opening and closing of skin pores and maintaining barrier function; the large intestine governs transmission, managing fluid metabolism and the elimination of waste. When lung qi is deficient, the defensive barrier weakens, compromising skin integrity and increasing susceptibility to external pathogens. Simultaneously, impaired lung qi descent can disrupt large intestine transmission, leading to stagnation of waste products and the internal generation of damp-heat. Conversely, stagnant damp-heat in the intestines can ascend to affect the lungs, further exacerbating skin lesions. This pathological connection between the “lung-intestine-skin” system aligns closely with the modern medical concept of the GSA (Zhang N. et al., 2025). The dialectical thinking of “observing the exterior to understand the interior” further demonstrates that skin symptoms on the body’s surface can reflect internal organ imbalances. This underpins the holistic therapeutic strategy of “treating skin (disease) by managing the intestine” (pi bing zhi chang) (Wang and Yihui, 2021). This synergistic effect of addressing both internal and external factors provides theoretical support for elucidating the pathological mechanisms of AD and expanding clinical diagnosis and treatment.

### 3.2 AM formula for treating AD

In TCM theory, AD is often associated with the accumulation of pathogenic factors such as wind, dampness, and heat in the skin. AM invigorates the spleen and augments qi to transform water-dampness, fortifies the lung, and consolidates the exterior defense to enhance the skin barrier. This aligns with the AD pathogenesis characterized by “spleen deficiency as the root, insecure lung defense, and damp-heat accumulation in the skin.” However, acute AD management prioritizes dispelling wind, clearing heat, and draining dampness to eliminate pathogenic factors. AM, with its qi-tonifying and exterior-consolidating actions, is more appropriate during remission, stable phases, or in AD patients with lung-spleen qi deficiency or defensive qi insecurity. Premature or excessive use of AM to “consolidate the exterior” during acute flares with exuberant damp-heat may trap pathogenic factors internally, hindering their expulsion and clearance. Therefore, in clinical practice, AM is frequently administered in conjunction with other botanical drugs. Therefore, clinical applications of AM often emphasize combination therapy to broaden its scope of use while fortifying the body’s defenses and expelling pathogens, thereby enhancing overall therapeutic efficacy.

Yupingfeng Powder (YPF-P), with AM as its sovereign medicinal agent, was first documented in the *Dan Xi Xin Fa* during the Yuan Dynasty (For specific formulation details, see Supplementary Appendix Table A1). Clinical studies demonstrate its significant efficacy in treating chronic urticaria, eczema, and other common dermatological conditions (Wang et al., 2020) and indicate potential for alleviating AD symptoms (Liu Y. X. et al., 2024). Clinical studies indicate that the combination of Yupingfeng Granules and cetirizine in treating infantile AD (spleen deficiency with dampness accumulation pattern) significantly increases the overall response rate after 3 weeks of treatment, reduces the EASI score, improves the CDLQI, and decreases the long-term recurrence rate (P < 0.05). This therapeutic advantage may be related to its immunomodulatory effects (Liu, 2019). Basic research indicates that YPF-P significantly inhibits type I hypersensitivity reactions, effectively reducing inflammatory responses in rat models of allergic skin diseases. Crucially, it does not induce the characteristic immunosuppressive side effects on immune organs associated with prednisone acetate-type drugs, with its mechanism linked to immune function regulation (Sun et al., 2018). During the relapse phase of AD, YPF-P demonstrates both short-term and long-term anti-allergic and anti-inflammatory effects by suppressing the production of key inflammatory mediators, including TNF-α, IL-6, IL-4, IL-5, and IgE. Its bioactive compounds (claycosin, formononetin, and cimifugin) also significantly improved skin barrier function in mouse models by repairing defects in tight junction proteins (Nie et al., 2024; Zheng et al., 2019). The Spleen-Nourishing, Blood-Nourishing, Wind-Dispelling Decoction is derived from the YPF-P, also with AM as the principal botanical drug (For specific formulation details, see Supplementary Appendix Table A2). Its functions include strengthening the spleen, dispelling wind, nourishing blood, and alleviating itching (Yang et al., 2021). This formula is a modern clinical empirical formula that is safe and effective in treating AD caused by blood deficiency and wind dryness (Wang et al., 2018). Studies show that it can regulate the abundance of *Bacteroides* in the gut, reduce serum IgE and IL-17A levels, increase transforming growth factor β1 (TGF-β1) and secretory IgA expression, repair the intestinal mucosal barrier, and thereby improve skin inflammation in mice with spleen deficiency-type AD (Chen et al., 2024).

TCM holds that “qi and blood share the same origin,” emphasizing that “the spleen is the source of qi and blood production.” Spleen deficiency impairs the effective conversion of nutrients from food into qi and blood. Blood possesses functions of nourishing yin and moistening dryness. Danggui Buxue Tang, a classic formula for tonifying qi and nourishing blood, is composed of AM and Angelica sinensis in a 5:1 ratio (Fang and Pan, 2024) (For specific formulation details, see Supplementary Appendix Table A3). The heavy dosage of AM aims to fortify the spleen and boost qi, enhancing the generation of qi and blood to moisten the entire body’s skin (Zhang et al., 2025b), thereby alleviating the AD pattern of blood deficiency with wind-dryness. Research has found that this compound formulation is more effective than using AM or Angelica sinensis extracts alone in significantly improving allergic-inflammatory reactions elicited in mouse models of AD. The underlying mechanisms involved suppression of serum IgE levels, reduction in mast cell infiltration, downregulation of key pro-inflammatory cytokines, and inhibition of NF-κB and MAPK inflammatory signaling pathway activation (Choi et al., 2016). A meta-analysis incorporating 37 clinical trials (n = 2973) demonstrated that TCM spleen-tonifying therapy for AD significantly outperformed conventional Western medication in improving both skin lesion severity (SCORAD score: WMD = −9.82) and itching symptoms (VAS score: WMD = −0.79) compared to conventional Western medication (both P < 0.00001). Furthermore, this therapy effectively modulates AD-related immune dysregulation (e.g., increasing IFN-γ and decreasing IL-4) and significantly reduces recurrence rates (OR = 0.36, P < 0.0001) (Liu et al., 2018). Notably, among the multiple spleen-tonifying formulas included in this meta-analysis, AM frequently appeared as a core ingredient. The effects of tonifying qi and consolidating the exterior, along with immune regulation, may represent a key mechanism underpinning the therapeutic efficacy of spleen-tonifying methods. In summary, multiple clinical studies have demonstrated that compound formulations with AM as the principal botanical ingredient significantly improve the severity of skin lesions (e.g., SCORAD and EASI scores), itching symptoms (as measured by VAS scores), and quality of life in AD patients, while also modulating immune markers. Although these effects result from the overall action of the compound, AM’s contribution as the core component responsible for tonifying qi, fortifying the exterior, and regulating immunity cannot be overlooked. This provides preliminary, directional clinical clues for the clinical development of AM monotherapy or its primary constituents.

### 3.3 Application of acupuncture in the treatment of AD

Modern medical research indicates that acupuncture can regulate human immune function by influencing T-cell activation processes and maintaining the dynamic equilibrium of Th1 and Th2 cytokine secretion, thereby improving the immune microenvironment in AD (Dai et al., 2018). Experiments in AD mice confirm that acupuncture suppresses the abnormally activated Type 2 immune response in AD, alleviates impaired epidermal barrier function, and exerts a restorative effect primarily by upregulating FLG expression in skin tissue and improving the ceramide metabolic pathway (Niu et al., 2025).In clinical practice, fire needling—a distinctive external treatment method in TCM—promotes blood circulation by warming and unblocking meridians, effectively alleviating the itching symptoms of AD caused by wind arising from blood deficiency. Chen et al.'s study demonstrated that the combination of moxibustion and loratadine tablets significantly reduced serum IL-4 and IgE levels in adult AD patients, improved disease scores such as SCORAD and EASI, enhanced quality of life (DLQI), achieved an overall response rate of 96.5%, and showed superior efficacy compared to the drug-only group (Chen et al., 2023a). Animal studies also support acupuncture’s barrier repair effects. Acupuncture reduces transepidermal water loss, IgE, and IL-17 levels in guinea pigs with eczema, increases stratum corneum hydration, and upregulates Aquaporin 3 protein expression in the lungs, skin, and rectum (Wang and Shen, 2022). Additionally, a randomized controlled trial demonstrated that verum acupuncture significantly outperformed sham acupuncture in improving SCORAD, VAS, EASI, POEM, and DLQI scores among patients with mild to moderate AD. Furthermore, no significant difference in efficacy was observed between twice-weekly and thrice-weekly acupuncture frequencies, indicating that acupuncture treatment exhibits good frequency adaptability and clinical feasibility (Kang et al., 2018).

## 4 Pharmacological Mechanisms of AM in AD treatment

### 4.1 Immune modulation

The characteristic immune dysregulation of AD manifests as Th2 dominance, Th17/Treg imbalance, and excessive release of pro-inflammatory factors. In the AD mouse model, local application of AM effectively suppressed the expression of Th2 cytokines (IL-4, IL-5, IL-6, IL-13) and TNF-α at AD lesion sites, regulated NF-κB signaling pathway activity, alleviated inflammatory cell infiltration, and improved epidermal hyperplasia (Kim et al., 2013). This suggests that AM has therapeutic potential for AD by regulating cytokine responses. Regulatory T (Treg) cells and Th17 cells exhibit dynamic antagonism in immune responses, specifically manifested as Treg cells suppressing inflammation and maintaining immune homeostasis, while Th17 cells promote autoimmunity and inflammatory responses (Lee, 2018; Zhang et al., 2021). APS and astragaloside restore the Th17/Treg immune equilibrium by suppressing Th17 cell differentiation and function while enhancing Treg cell proliferation and activity (Huang et al., 2024). In pathological conditions mediated by Th2-type immune responses, approximately 80% of AD patients have significantly elevated serum IgE levels, and IgE is the core molecule that drives the activation of effector cells (such as mast cells and basophils) in allergic inflammation (Kim et al., 2022; Shamji et al., 2021). In addition, these two components can significantly inhibit IgE expression, downregulate pro-inflammatory factors, and simultaneously upregulate the anti-inflammatory factor IL-10, thereby synergistically correcting immune imbalance and alleviating allergic symptoms (Liu L. Y. et al., 2023; Zhang S. J. et al., 2022; Zhong et al., 2022). In allergic reactions, mast cell degranulation triggers immediate hypersensitivity reactions, which are then followed by macrophage-mediated inflammatory responses. Studies have revealed that AM leaf extract (AMLE) has a stronger inhibitory effect than AM root extract. Among these, quercetin and kaempferol, as the main polyphenolic active components in AMLE, can effectively inhibit the release of inflammatory mediators in immune cells, thereby regulating allergic immune responses and inflammatory processes (Bunddulam et al., 2025). Further research indicates that AS-IV effectively alleviates symptoms of atopic diseases such as asthma, allergic rhinitis, and AD by regulating Th1/Th2 immune skewing and shows potential for blocking the atopic process (Yu et al., 2022).

### 4.2 Anti-inflammatory effect

The core driver of the AD inflammatory cascade involves epithelial-derived cytokines, including IL-33, IL-25, and TSLP, which stimulate group ILC2 to produce type 2 cytokines, thereby amplifying and perpetuating the inflammatory response. Studies indicate that prophylactic administration of AS-IV during the sensitization phase can mitigate inflammatory responses in a mouse model of allergic contact dermatitis by suppressing the expression of the initiator cytokines TSLP and IL-33 and inhibiting the activation of ILC2 cells (Bao et al., 2016). Research evidence from cell and animal studies indicates that total flavonoids of AM possess immunomodulatory and anti-inflammatory effects. The core mechanism involves inhibiting the activation of key inflammatory signaling pathways such as MAPK, NF-κB, upregulating the mRNA levels of the anti-inflammatory factor IL-10, and broadly suppressing the expression of pro-inflammatory factors TNF-α, IL-1β, IL-6, IFN-γ, as well as inflammatory enzymes inducible nitric oxide synthase (iNOS) and cyclooxygenase-2 (COX-2) (Guo et al., 2016; Li J. et al., 2018). Different flavonoids exert differential effects by targeting specific pathways. Formononetin alleviates AD pathology by suppressing epithelial-derived TSLP/IL-33 release via E-cadherin modulation. It concurrently inhibits NF-κB, MAPK, JAK-STAT signaling, and NLRP3 inflammasome activity, reducing proinflammatory mediators including TNF-α, IL-6, and COX-2/PGE2 (Ding et al., 2024; Li L. et al., 2018). Furthermore, its phytoestrogenic properties activate G protein-coupled estrogen receptor (GPER), upregulating A20 while downregulating TSLP (protein and mRNA levels), demonstrating synergistic anti-inflammatory effects in AD skin (Yuan et al., 2021). The *in vivo* study further demonstrated that Formononetin significantly improved the typical pathological features, such as erythema and epidermal thickening, in a mouse model of psoriasis, and the mechanism involved the downregulation of IFN-γ and IL-17 (Xu et al., 2024). Calycosin regulates the Treg/Th17 balance, inhibits the expression of factors such as IL-4, IL-5, IL-13, TSLP, and IL-33, and suppresses the activation of the NF-κB pathway, effectively alleviating allergic inflammation in mice and restoring epithelial barrier function (Ma et al., 2024; Yu et al., 2017). Kaempferol improves AD-like dermatitis and repairs the skin barrier by inhibiting type 2 inflammatory responses, TSLP expression, and oxidative stress, demonstrating therapeutic potential (Nasanbat et al., 2023). Additionally, kaempferol alleviates excessive proliferation of keratinocytes by inhibiting the IFN-γ-induced JAK-STAT signaling pathway. Simultaneously, it reduces the number of γδT17 cells and suppresses the expression of key inflammatory cytokines such as IL-17, IL-23, and TNF-α, thereby effectively alleviating psoriatic lesions (Li et al., 2023). APS exerts anti-inflammatory effects by inhibiting the MAPK/NF-κB signaling pathway and reducing the expression of inflammatory factors (Chen et al., 2023b; Dong et al., 2020). Its anti-inflammatory activity can be significantly enhanced through honey roasting (Liao et al., 2018). The compound isolated by Kim et al. from the aqueous methanol layer of AM exhibited potent anti-inflammatory effects in a psoriasis-like mouse model. The mechanism involved the inhibition of reactive oxygen species (ROS) generation, blockade of NF-κB, JAK, and STAT signaling pathways, and suppression of pathogenic Th17/Th2 T cell differentiation (Kim et al., 2014). NLRP3 inflammasomes play a key role during both the acute and chronic stages of AD by regulating Th2/Th1 immune bias, and their pharmacological inhibition has the potential to alleviate AD pathology (Sun et al., 2025). Complanatuside, derived from AM, also holds potential therapeutic value for skin inflammation. This compound effectively reduces pyroptosis and cell death by inhibiting the NLRP3 inflammasome pathway, thereby downregulating pyroptosis-associated proteins and inflammatory mediators, thus protecting skin cells from inflammatory damage (Wang J. et al., 2022).

### 4.3 Promotes skin damage repair

Epidermal barrier defects represent a central pathological mechanism underlying the emergence and worsening of AD. AM and its bioactive components demonstrate significant therapeutic efficacy in promoting skin tissue repair and barrier function reconstruction through a multi-target regulatory mechanism. Research has confirmed that AM can alleviate the severity of DNFB-induced AD-like skin lesions in mice by inhibiting the production of IFN-γ by CD4^+^ T cells (Lee et al., 2007). In addition, Derma-Hc, a traditional Korean herbal formula centered on AM, can effectively inhibit AD-related skin lichenification, itching, epidermal hyperplasia, and hyperkeratosis (Jo et al., 2020; Nam et al., 2021). Tight junction forms a continuous barrier that restricts the entry of allergens, microorganisms, and irritants while also modulating TEWL (Kirschner et al., 2013). However, in AD, type 2 inflammatory responses impair this barrier function. This impairment manifests as reduced expression or dysfunction of claudin proteins, triggering a vicious cycle: increased inflammation, enhanced allergen penetration, and greater susceptibility to infection (Beck et al., 2022; Katsarou et al., 2023). The study by Jia et al. revealed that calycosin alleviates allergic skin inflammation by enhancing the expression and distribution of tight junction proteins, thereby restoring epidermal barrier function and reducing Th2-type immune responses (Jia et al., 2018). Hypertrophic scars are an abnormal outcome of tissue repair after trauma, resulting from the overgrowth of fibroblasts coupled with excessive extracellular matrix deposition (Han et al., 2017). Intense pruritus in AD patients leads to repeated deep scratching, causing skin lesions that significantly elevate the risk of pathological scarring (Kwon et al., 2021; Limmer et al., 2023). Formononetin arrests human proliferative scar fibroblasts in the G1/S phase by inhibiting Cyclin D1 expression and induces apoptosis by downregulating Bcl-2 expression, thereby regulating scar growth (Hui et al., 2019). E-cadherin is a key adhesion molecule that maintains epithelial homeostasis (Lialios and Alimperti, 2025). In the AD model, quercetin improves wound healing disorders in keratinocytes by upregulating the expression of claudin and E-cadherin, inhibiting abnormal MMP activity, and regulating the extracellular signal-regulated kinase 1/2/mitogen-activated protein kinase ERK1/2 MAPK and NF-κB pathways through its anti-inflammatory and antioxidant properties (Beken et al., 2020). In addition, quercetin can inhibit the proliferation of keloid fibroblasts by suppressing the STAT3/p-STAT3 signaling pathway, thereby exerting an anti-fibrotic effect (Fu et al., 2020). Keratinocytes in AD serve as both initiators and promoters of the inflammatory response and guardians of the skin barrier (Das et al., 2022). Abnormal differentiation of AD skin keratinocytes leads to excessive proliferation of the epidermal basal layer, inhibition of terminal differentiation markers, abnormal barrier lipids, and damage to the skin barrier and antimicrobial function (Goleva et al., 2019). Nguyen et al. found that AM and its compound formononetin can protect skin from barrier damage caused by environmental factors by inhibiting the ERK pathway and regulating the proliferation and apoptosis of keratinocytes, thereby restoring epidermal integrity (Nguyen et al., 2019). Zhao et al. showed that APS can facilitate the proliferation of human cutaneous fibroblasts. The mechanism involves suppressing inflammation, accelerating the cell cycle process, and stimulating the secretion of repair factors, indicating its potential in wound healing (Zhao et al., 2017). Additionally, studies have found that AS-IV and its primary metabolite, cycloastragenol-6-O-β-D-glucoside, activate the epidermal growth factor receptor/mitogen-activated protein kinase (EGFR/ERK) signaling pathway, significantly accelerating the healing of sterile and infected wounds in mouse models, thereby promoting the proliferation, migration, and angiogenesis of keratinocytes and fibroblasts (Gao et al., 2024).

### 4.4 Antioxidant stress response

Persistent inflammatory responses and impaired epidermal barrier function in AD drive excessive ROS generation, resulting in oxidative stress (OS). OS, in turn, activates pro-inflammatory pathways (such as NF-κB), disrupts the skin barrier, and exacerbates immune imbalance, forming a self-perpetuating vicious cycle that collectively drives the onset and progression of AD (Bertino et al., 2020; Borgia et al., 2022; Choi et al., 2021; Nakai and Tsuruta, 2021). AM, a medicinal botanical drug rich in natural antioxidants, contains multiple active components that intervene in OS through multiple pathways: directly scavenging free radicals, enhancing the activity of the endogenous antioxidant enzyme SOD, inhibiting the activity of pro-oxidative enzymes, reducing lipid peroxidation, chelating metal ions, and regulating key signaling pathways (Yao et al., 2024). The active constituents of AM, methylnissolin-3-O-β-D-glucopyranoside and formononetin, mitigate endothelial OS injury and exert protective effects by activating the Nrf2/HO-1 pathway to scavenge ROS (Ding et al., 2024; Wu et al., 2021). The antioxidant stress effects of APS are mainly manifested in enhancing the endogenous antioxidant defense system, inhibiting lipid peroxidation, and antagonizing UVB-induced oxidative damage to keratinocytes through photoprotective effects (Li et al., 2016; Yan et al., 2010). AS-IV can reduce ROS levels, inhibit TLR4 and its downstream NF-κB, iNOS, and COX-2 protein expression, alleviate UVB-induced keratinocyte oxidative damage, inflammatory response, and apoptosis, and ultimately enhance cell vitality (Wang et al., 2022b). Quercetin enhances antioxidant defense by activating Nrf2 and inhibits pro-oxidative pathways, specifically by blocking NF-κB nuclear translocation to reduce TNF-α and IL-1β production, while also inhibiting NOX2 expression by activating HO-1. Additionally, quercetin regulates the PI3K, AMPK, and MAPK signaling pathways to collectively alleviate OS and maintain oxidative balance (Zhou et al., 2023).

**FIGURE 2:**
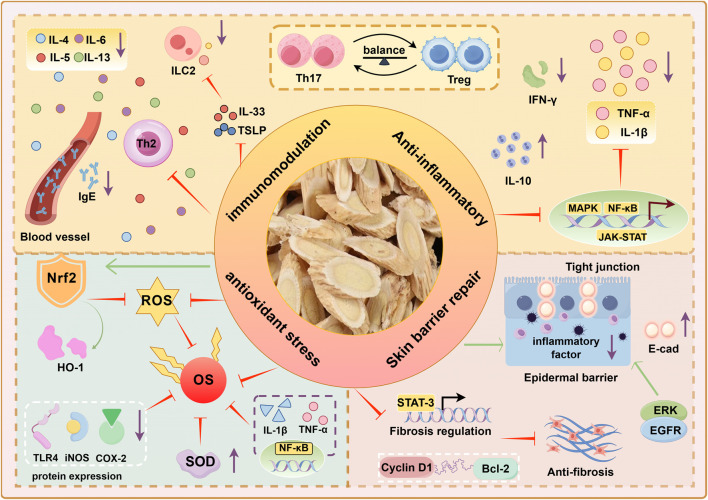
Pharmacological mechanisms of AM in AD treatment.

Overall, the mechanisms by which AM treats AD primarily involve modulating immune dysregulation, exerting anti-inflammatory effects, promoting skin barrier repair and wound healing, and effectively counteracting oxidative stress (Figure 2). These combined actions collectively ameliorate the immunological abnormalities, chronic inflammation, skin barrier defects, and oxidative stress vicious cycle in AD, demonstrating significant therapeutic potential.

In terms of immune regulation and anti-allergic effects: By regulating the balance of Th17/Treg cell differentiation, inhibiting pro-inflammatory factors such as IL-17 and IL-6, and upregulating anti-inflammatory factors like IL-10, immune homeostasis is restored. Blocking the secretion of Th2 cytokines and TNF-α, inhibiting IL-33/TSLP-mediated ILC2 activation, and reducing allergic reactions. Regulates inflammatory pathways such as NF-κB and MAPK, and reduces serum IgE expression. In promoting wound healing, it activates growth factor signaling, accelerates fibroblast proliferation and migration, and promotes re-epithelialization. Enhances E-cadherin and claudin expression, inhibits MMP activity, and maintains epithelial integrity. In terms of antioxidant stress, it scavenges ROS, activates the Nrf2 pathway to enhance antioxidant defense, and suppresses NF-κB-driven inflammation, thereby disrupting the oxidative stress-inflammation cycle in AD.

Nrf2: Nuclear factor erythroid 2-related factor 2, ROS: Reactive oxygen species, OS: Oxidant Stress, SOD: Superoxide Dismutase, HO-1: Heme Oxygenase-1, NQO1: NADH Quinone Dehydrogenase 1, COX-2: Cyclooxygenase-2, iNOS: Inducible Nitric Oxide Synthase, E-cad: E-cadherin, EGFR: Epidermal Growth Factor Receptor, ERK: Extracellular Signal-Regulated Kinase, Bcl-2:B-cell lymphoma-2.

## 5 Gut-Skin-Axis

### 5.1 Relationship between AD and the GSA

Intestinal flora and their metabolites profoundly affect skin health through the GSA, which contributes significantly to the pathogenesis of AD. In a healthy state, the gut microbiota maintains homeostasis by shaping gut-associated lymphoid tissues (GALT)—central to mucosal immunity—restricting commensal bacterial translocation, and mediating immune signaling from the gut to the skin (Mahmud et al., 2022). However, in individuals susceptible to AD, impaired intestinal epithelial barrier integrity and dysfunction of tight junction proteins frequently occur, leading to abnormally elevated intestinal permeability (i.e., the “leaky gut” phenomenon). This pathological state facilitates the translocation of enteric bacteria, viruses, and their metabolic byproducts into the lamina propria, thereby activating adaptive immune responses. This triggers the massive release of inflammatory cytokines, which may further enter the systemic circulation, causing systemic effects (Martens et al., 2018; Mohammad et al., 2024; Rios-Carlos et al., 2024). Subsequently, circulating metabolites and toxins accumulate in cutaneous tissues. There, they induce a Th2-type immune response and release pro-inflammatory mediators, thereby disrupting skin homeostasis (Song et al., 2016).

SCFAs are the primary metabolites produced by bacterial fermentation of dietary fiber in the gastrointestinal tract, playing a crucial role in maintaining intestinal homeostasis and regulating immune responses (Liu S. et al., 2023). Specifically, SCFAs can effectively reduce the risk of systemic inflammation by enhancing intestinal barrier function and minimizing the entry of lipopolysaccharides (LPS) into the bloodstream. Simultaneously, they exert bidirectional regulation at the immune cell level: on one hand, inducing naive T cells to differentiate into anti-inflammatory regulatory T cells (Tregs); on the other hand, suppressing pro-inflammatory factors like TNF-α and IL-6 produced by intestinal macrophages, while further inhibiting IL-8 production by normal intestinal epithelial cells (IECs), thereby effectively alleviating intestinal inflammation (Yao et al., 2022; Zhang et al., 2023). SCFAs, as key signaling molecules in the GSA, contribute to skin barrier homeostasis by enhancing keratinocyte turnover and maturation, thereby alleviating skin inflammation (Trompette et al., 2022; Xiao et al., 2022). Further research indicates that SCFAs (such as butyrate) act as activators of the G protein-coupled receptors GPR43 and GPR109a, promoting anti-inflammatory effects and thereby maintaining intestinal homeostasis (Krejner et al., 2018; Van der Hee and Wells, 2021). In AD patients, SCFAs exhibit abnormalities characterized by significantly lower fecal concentrations compared to healthy individuals, accompanied by a reduction in the abundance of SCFA-producing bacteria (Barman et al., 2024; Reddel et al., 2019). Therefore, the deficiency of SCFAs weakens their regulatory role in the intestinal barrier and immune function, which may be a key mechanism through which the GSA exacerbates AD skin inflammation.

### 5.2 Mechanism of AM regulating the GSA in the treatment of AD

The proposal of the GSA has opened up new avenues for targeted interventions in the prevention and treatment of AD (Fang et al., 2021). Clinical studies indicate that the onset and progression of AD are closely associated with an imbalance in gut microbiota composition. This imbalance is characterized by a decrease in the abundance of beneficial bacterial genera such as *Lactobacillus* and *Bifidobacterium*, coupled with an increase in the proportion of potentially pathogenic bacteria, including *Escherichia coli*, *Clostridium difficile*, and *S*.*aureus*. (Yap et al., 2014). APS and AS-IV both exhibit dual regulatory effects on the intestinal microbiota: on one hand, they promote the proliferation of beneficial bacteria like *Lactobacillus* and *Bifidobacterium*; on the other hand, they inhibit the growth of harmful bacteria such as *E. coli* and *Salmonella*, thereby restoring the balance of the intestinal microbiota (Lyu et al., 2024; Wen et al., 2024; Zhang Y. et al., 2022). Importantly, the proliferated *Lactobacillus* and *Bifidobacterium* strains can produce SCFAs through the fermentation of dietary fiber, thereby significantly elevating SCFAs levels within the gut. This plays a pivotal role in enhancing intestinal barrier function and regulating immune homeostasis (Markowiak-Kopeć and Śliżewska, 2020). In the regulation of the GSA, impaired intestinal barrier function is considered a pivotal component initiating a vicious cycle. Research indicates that both AM and its active component AS-IV can significantly improve intestinal barrier dysfunction in rats with ulcerative colitis by regulating gut microbiota homeostasis and mitigating inflammatory damage (Zhang et al., 2024; Zhu et al., 2025). APS enhances intestinal barrier function by strengthening physical, biochemical, and immune barriers. They achieve this through: Increasing expression of tight junction proteins (e.g., ZO-1, Occludin, Claudin-1), suppressing OS levels, promoting growth of beneficial microbiota, and increasing SCFAs production, modulating immune responses (e.g., elevating sIgA levels) (Chen et al., 2022; Jia et al., 2025; Liang et al., 2024). Multiple model experiments have confirmed that APS exhibits significant prebiotic activity, effectively improving intestinal microecological imbalance and associated immune dysregulation. Its mechanisms of action include increasing gut microbiota diversity, promoting SCFAs production, inhibiting the TLR4/NF-κB inflammatory signaling pathway, and regulating Th17/Treg cell homeostasis (Rong and Shu, 2024; Song et al., 2022; Wei et al., 2023; Zhang et al., 2025c; Zhao et al., 2023). Similarly, kaempferol also restores gut microbiota diversity and inhibits the LPS-TLR4-NF-κB signaling pathway, thereby improving intestinal barrier function and suppressing inflammatory responses (Qu et al., 2021). Additionally, AM and its active components can effectively alleviate intestinal inflammation through anti-inflammatory mechanisms, thereby maintaining the integrity of intestinal epithelial cells. Its primary actions include inhibiting the NF-κB signaling pathway, reducing the expression of pro-inflammatory factors such as TNF-α, COX-2, and iNOS, and activating Nrf2-mediated antioxidant pathways, thus synergistically exerting intestinal protective effects (Adesso et al., 2018; Cui et al., 2018; Lv et al., 2017).

**FIGURE 3:**
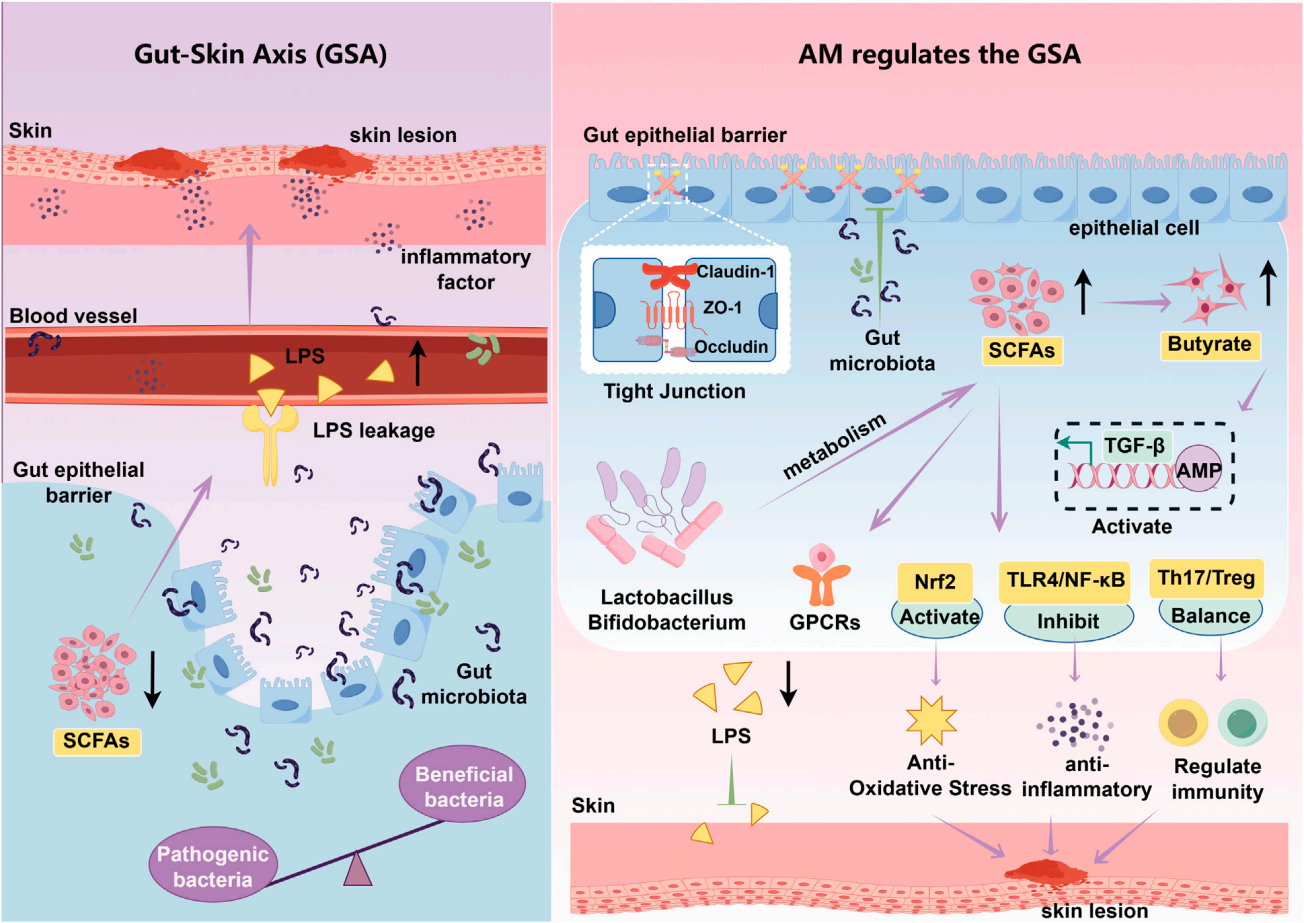
Mechanism of APS intervention in AD via the GSA. Lps: Lipopolysaccharide, TGF-β:Transforming growth factor-β, AMP: Adenosine monophosphate, GPCRs: G protein-coupled receptors, TLR4: Toll-like receptor 4. Purple arrows indicate promotion, while green T-shaped icons indicate inhibition. Black arrows pointing upward represent an increase, while those pointing downward represent a decrease.

In summary, based on the GSA theory, the core mechanism of AM in treating AD lies in its active components reshaping the gut microbiota structure and promoting the production of SCFAs, primarily butyrate (Figure 3). This enhances intestinal barrier function and regulates immune homeostasis. This process effectively suppresses endotoxin translocation and abnormal immune activation, blocking the transmission of inflammatory signals to skin tissues. This demonstrates AD’s therapeutic advantage of exerting multi-targeted, holistic regulation through the GSA.

## 6 Computer prediction and simulation

### 6.1 Main active ingredients and targets of AM

Using the TCM Systems Pharmacology Database and Analysis Platform (https://www.tcmsp-e.com/), we searched and initially identified 87 compounds from AM. These were further screened based on the criteria of oral bioavailability (OB) ≥ 30% and drug-like properties (DL) ≥ 0.18 (Aungst, 2017; Jia et al., 2020). However, we note that strict OB/DL criteria may exclude certain key pharmacodynamic components with clear pharmacological efficacy but lower oral bioavailability or lower pharmacokinetic scores. To ensure network pharmacology analysis comprehensively covers the material basis of AM treatment for AD, we supplemented two components—AS-IV and Linolenic Acid—based on the following rationale: AS-IV serves as the core quality indicator for AM in the Chinese Pharmacopoeia. Studies have demonstrated its ability to significantly improve allergic conditions such as AD and asthma by regulating Th17/Treg balance and inhibiting key inflammatory mediators (Liang et al., 2023; Yu et al., 2022). α-linolenic acid (ALA) is an essential omega-3 polyunsaturated fatty acid for the human body. It serves as the metabolic precursor for longer-chain omega-3 fatty acids within the body. Research indicates that supplementation with long-chain omega-3 fatty acids may reduce the risk and severity of AD through anti-inflammatory and immunomodulatory mechanisms (Reese and Werfel, 2015). Furthermore, in an ovalbumen-induced allergic rhinitis mouse model, ALA has been demonstrated to significantly alleviate inflammatory responses and allergic symptoms by regulating the Th1/Th2 cell balance and the JAK/STAT signaling pathway (Ren et al., 2022). Dietary supplementation with γ-linolenic acid (GLA) can be converted into anti-inflammatory metabolites that suppress skin inflammation and enhance barrier function. This reduces transepidermal water loss (TEWL), improves skin hydration levels, and alleviates itching symptoms (Kawamura et al., 2011). Therefore, AS-IV and alpha-linolenic acid were included as candidate compounds to establish a more comprehensive pharmacological basis for AM in treating AD. After screening, a total of 21 natural chemical constituents of AM were ultimately included for subsequent research (see Supplementary Appendix Table A4). The target proteins for these active components were obtained through the Swiss TargetPrediction platform (http://www.swisstargetprediction.ch). The species was set to “*Homo sapiens*”. After deduplication and integration, a total of 491 standard gene targets were ultimately obtained.

### 6.2 AD target screening and intersection target acquisition

**FIGURE 4:**
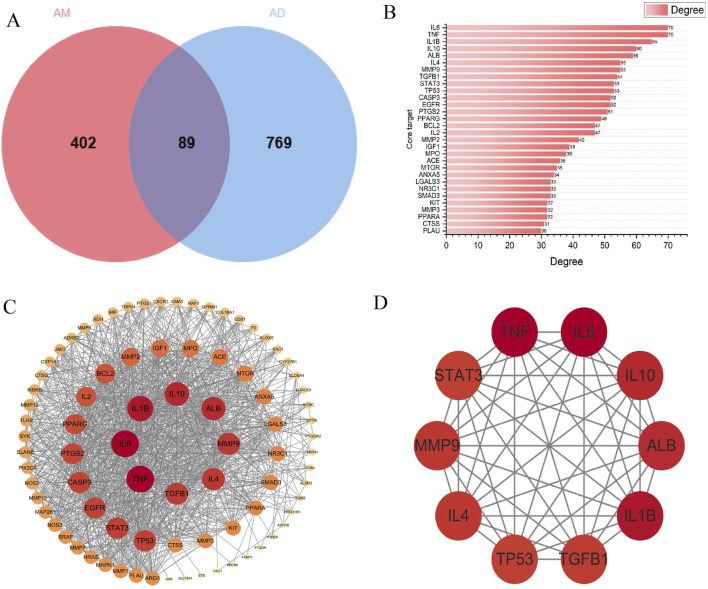
Core AM-AD Targets and PPI Network Analysis. **(A)** Venn diagram of AM and AD intersection targets. The circle on the left corresponds to 491 AM-related targets, while the circle on the right represents 858 AD-related targets. The overlapping area indicates 89 overlapping therapeutic targets. **(B)** The top 30 targets ranked by connectivity in the PPI network. **(C)** PPI network of common targets. Circular nodes denote proteins, while connecting edges represent protein-protein interactions. Node dimensions and chromatic intensity reflect both degree values and target relevance, where enlarged nodes with intensified coloration indicate higher centrality of critical targets. These targets occupy hub positions within the network topology, suggesting their essential roles in AD therapeutic mechanisms. **(D)** Top 10 core targets within the PPI network.

Search GeneCards (https://www.genecards.org/), TTD (https://db.idrblab.net/ttd/), and OMIM (https://www.omim.org/) using the search term “atopic dermatitis”, yielding 816, 64, and 12 AD targets, respectively. After merging and deduplication, 858 unique AD targets were obtained. The screened AM targets and AD targets were imported into the Venn platform (https://www.bic.ac.cn/test/venn/#/), yielding 89 intersection targets, which were visualized using a Venn diagram (Figure 4A). Key targets with a degree value exceeding 30 were visualized in a bar chart (Figure 4B).

### 6.3 PPI network construction and key target screening

To obtain protein interaction relationships, we submitted the screened intersection targets to the STRING platform (https://string-db.org/) to construct a PPI network. Import the PPI network diagram into Cytoscape (Version 3.10.0) for visualization, hiding nodes disconnected by network breaks while retaining all other parameters at default settings. The final PPI network diagram comprises 89 nodes and 1093 edges (Figure 4C). Using the CytoHubba plugin to calculate the degree of targets in the PPI network, it is noteworthy that multiple targets in the network (such as IL-6, TNF-α, IL-1β, etc.) occupy central positions with high node degree values. This suggests that these targets may play pivotal roles in the process of AM intervention in AD. Finally, the top 10 core targets with the highest degree values were selected (Figure 4D). The values, from highest to lowest, were: IL-6, TNF-α, IL-1β, IL-10, ALB, IL-4, MMP-9, TGF-β1, STAT-3, TP53. These targets may play important regulatory roles in AM treatment for AD (Table 1).

**TABLE 1:** Key hub targets screened by degree centrality and their functional characteristics in the treatment of AD.

Target	Degree	Type	Primary functions and mechanisms of action	References
IL-6	70	Pro-inflammatory cytokines	1. Induces the expression of acute inflammatory proteins such as C-reactive protein 2. Activates Th17 cells, suppresses Treg cells, and mediates inflammatory responses	Jordan et al. (2017), Uciechowski and Dempke (2020)
TNF-α	70	Pro-inflammatory cytokines	1. Mediates the development of inflammation and autoimmune disorders 2. Induction of epithelial cell death 3. AD biomarkers	Andrews et al. (2018), Jang et al. (2021), Kalashnikova et al. (2024)
IL-1β	65	Pro-inflammatory cytokines	1. Exacerbates the inflammatory process in AD 2. Recruits and activates immune cells 3. Impairs skin barrier function and synergistically enhances allergen sensitization intensity with TSLP 4. Promotes Th2/Tfh differentiation and subsequent asthma development	Alshevskaya et al. (2016), Bernard et al. (2017), Segaud et al. (2022), Sikorska-Szaflik et al. (2025)
IL-10	60	Anti-inflammatory cytokines	1. Induces anti-inflammatory mediators, suppresses Th1 responses, and promotes Treg function 2. Inhibits eosinophil activation, infiltration, and degranulation, reducing the severity of AD inflammation	Lee et al. (2024), Saraiva et al. (2019)
IL-4	55	Th2 cytokines	1. Activates Th2-polarized function of dendritic cells to drive allergic inflammation 2. Induces skin barrier dysfunction 3. Promotes IgE-mediated sensitization reactions	Leyva-Castillo et al. (2024), Torres et al. (2025)
MMP-9	55	Matrix metalloproteinase	1. degrades extracellular matrix 2. participates in tissue remodeling and wound healing 3. disrupts mucosal barriers 4. promotes inflammatory cell migration	Bayer et al. (2020), Sosna et al. (2023), Wang et al. (2024)
TGF-β	54	Immunosuppressive Cytokines	1. Induces Treg cell differentiation, suppress Th1/Th2 responses, and regulate immune responses 2. Promotes inflammatory responses and tissue fibrosis	Araujo et al. (2020), Shafi et al. (2024)
STAT-3	53	Transcription factor	1. Regulates IL-31 expression, participating in the development of inflammatory pruritus 2. Integrates pro-fibrotic signals 3. Bidirectionally modulates immune responses	Chakraborty et al. (2017), Hillmer et al. (2016), Takahashi et al. (2023)

### 6.4 GO enrichment and KEGG pathway analysis

**FIGURE 5:**
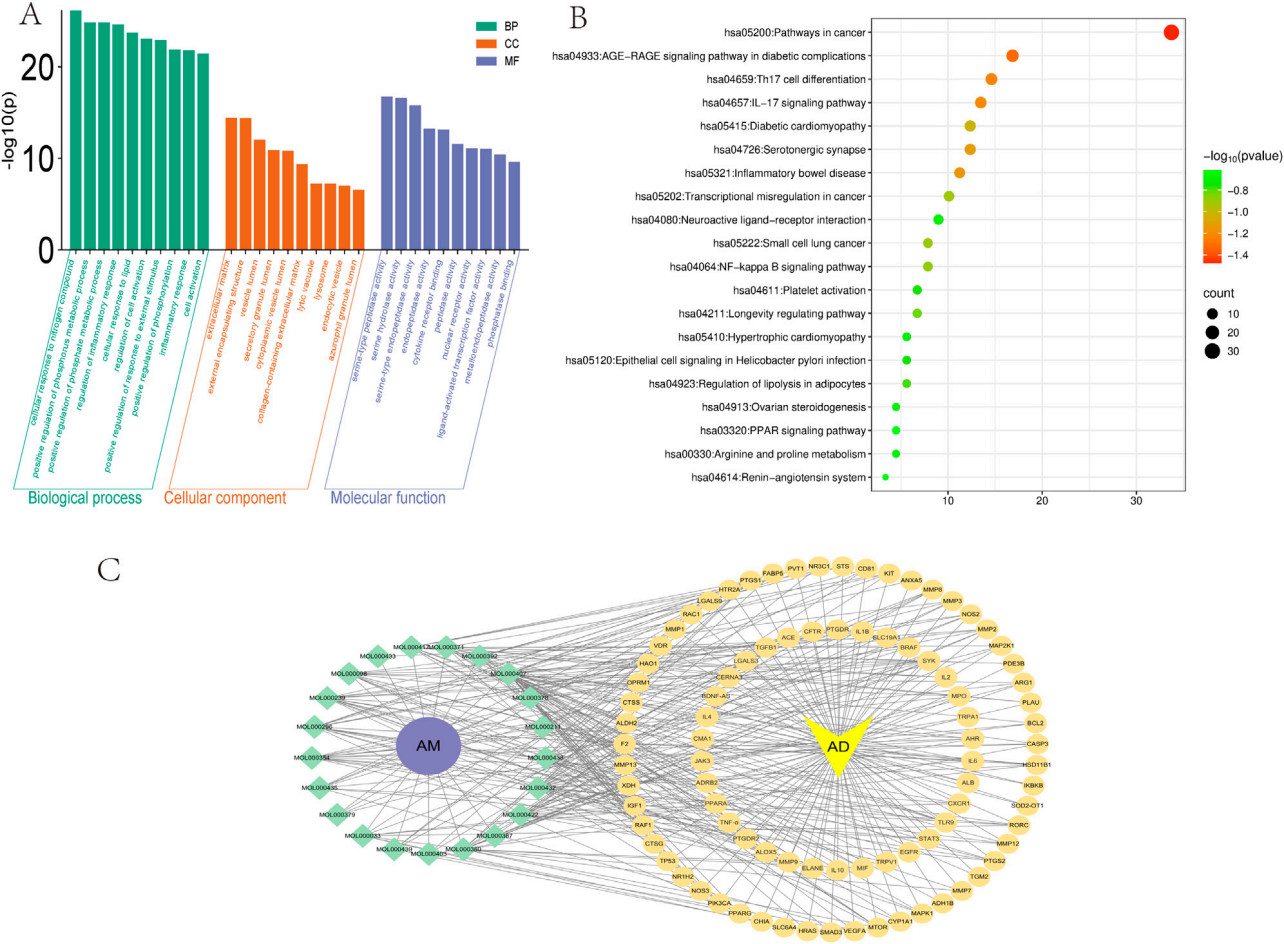
GO, KEGG enrichment analysis, and active ingredient-target interaction network diagram **(A)** GO enrichment analysis of AM and AD includes three categories: BP, CC, and MF. **(B)** KEGG analysis of AM and AD. Node size corresponds to target gene count per pathway, while color indicates statistical significance (-log10(p)). **(C)** Intersection target interaction network diagram. The yellow circle represents the common target gene of AM and AD. The yellow inverted triangle represents the disease AD. The green diamond represents the ID of the active ingredient of AM that meets the screening criteria. The purple circle represents AM. This line plot illustrates the compound-target binding relationship.

The intersection targets were imported into the Metascape platform (Zhou et al., 2019) (http://metascape.org/gp/index.html) for GO functional enrichment analysis (Limit to species “*Homo sapiens*,” set P < 0.01) (Consortium, 2021) and KEGG pathway enrichment analysis (Yuan et al., 2022). Visualization was performed using the MicroBioinformatics platform (http://www.bioinformatics.com.cn). In the GO functional enrichment analysis, 1,357 molecular biological processes (BP), 58 cellular components (CC), and 133 molecular functions (MF) were identified. The larger the -log10 value, the more genes were enriched. Finally, the top 10 entries for BP, CC, and MF were selected and plotted in a bar chart (Figure 5A). GO enrichment analysis implicated BP in the coordination of inflammatory and immune responses, potentially involving the modulation of Th2/Th17 differentiation imbalance and the suppression of pro-inflammatory cytokines (e.g., IL-6, TNF-α). CC alterations were primarily associated with the extracellular matrix, encompassing the restoration of barrier integrity mediated by molecules such as E-cadherin and occludin. MF significantly implicated protease activity and substrate hydrolysis, alongside molecular interactions and signaling regulation, notably manifesting as the inhibition of MMP activity. The intervention mechanism of AM in AD may involve biological processes such as inhibiting inflammatory signaling pathways, regulating immune homeostasis, and promoting skin barrier repair. The enrichment analysis results provide a potential molecular basis and predictive evidence for multi-target synergistic effects in this direction.

KEGG enrichment analysis of pathways revealed 190 signaling routes, where the top 20 entries ranked by abundance were chosen for bubble chart display (Figure 5B). The results revealed that AM-mediated therapeutic effects on AD involve critical pathways, including Th17 cell differentiation, IL-17 signaling pathway, and NF-κB signaling pathway. As extensively discussed in previous reviews, the NF-κB pathway has long been recognized as a classical pro-inflammatory signaling cascade (Lawrence, 2009). APS has been shown in multiple studies to effectively inhibit the activation of NF-κB and its downstream signaling pathways. Experimental evidence indicates that APS exerts significant anti-inflammatory effects in various inflammatory disease models (such as sodium dextran sulfate-induced colitis, allergic rhinitis, and LPS-induced neuroinflammation) by inhibiting the NF-κB pathway (He et al., 2022; Liu J. F. et al., 2025; Lv et al., 2017). This further highlights the key role of APS regulation of the NF-κB pathway in anti-inflammatory treatment. The lesional skin of individuals with AD is characterized by elevated IL-17 expression relative to healthy skin. As the primary cellular source of IL-17, Th17 cells have been established as key contributors to the pathogenesis of AD. Consequently, targeting specific bacterial species within gut dysbiosis to modulate Th17 cell activity may emerge as a novel therapeutic strategy for AD and related diseases (Yasuda et al., 2019).

### 6.5 Active ingredient-intersection target network construction

Using Cytoscape (Version 3.10.0) software, we constructed an “AM bioactive components-intersecting targets-pathways” network diagram (Figure 5C), systematically elucidating the multi-component, multi-target, and multi-pathway synergistic characteristics of AM in treating AD. This network encompasses 89 intersecting targets, multiple active compounds (e.g., AS-IV, calycosin, kaempferol), and several key signaling pathways (e.g., IL-17, NF-κB, TNF-α). Network topology analysis revealed high connectivity density, suggesting potential synergistic or complementary interactions among AM bioactive components in jointly regulating AD-related inflammation, immunity, and barrier function.

The network exhibits “one compound-multiple targets” and “one target-multiple compounds” phenomena. For instance, AS-IV simultaneously targets multiple core molecules, including IL-6, TNF-α, and IL-1β, while IL-6 itself is co-regulated by various AM components. This many-to-many interaction pattern validates, at the network level, AM’s holistic regulatory advantage in synergistically treating AD through its “multi-component, multi-target, multi-pathway” approach. The network not only visually illustrates the intricate connections between AM’s active components and key AD targets but also provides a visual tool and predictive framework for further exploration of its mechanisms of action.

### 6.6 Molecular docking

**FIGURE 6:**
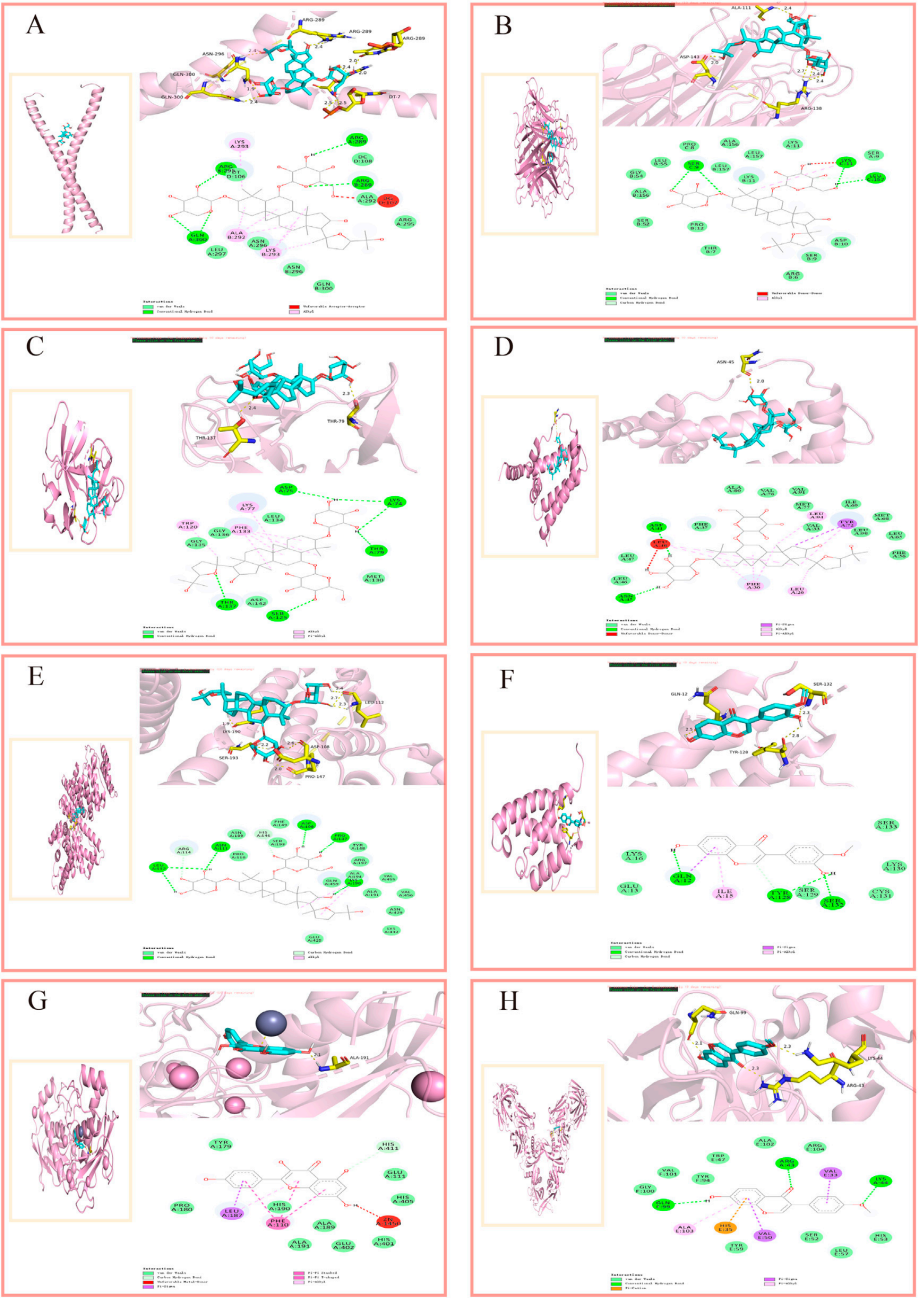
Molecular docking interaction diagrams. **(A)** AS-Ⅳ/IL-6, **(B)** AS-Ⅳ/TNF-α, **(C)** AS-Ⅳ/IL-1β, **(D)** AS-Ⅳ/IL-10, **(E)** AS-Ⅳ/ALB, **(F)** calycosin/IL-4, **(G)** kaempferol/MMP-9, **(H)** formononetin/TGF-β1.

Molecular docking, as a key tool in structural biology and drug discovery, enables efficient screening of new ligands by predicting the three-dimensional binding conformations and binding energies of compounds and targets (Paggi et al., 2024). To evaluate the binding potential between the active components of AM and its core targets, we conducted this study. The 2D structures of the core components were downloaded from the PubChem database (https://pubchem.ncbi.nlm.nih.gov/), converted into 3D structures using ChemBioOffice Ultra1 (Version 3.0.2) - Chem3D software, and optimized to minimize energy. Download the protein structure corresponding to the core target from the PDB database (https://www.pdbus.org/) (Burley et al., 2017) and use PyMOL (Version 2.5.2) software to perform dehydration and residue removal. In the AutoDockTools (Version 1.5.7) software, set the docking grid points and dimensions to ensure the docking box fully encloses the protein. Semi-rigid molecular docking was performed using Vina (Version 1.1.2) software. Each ligand was run 10 times, outputting the binding energy (kcal·mol^-1^) for the best conformation. Binding energy is a crucial metric for evaluating ligand-target interactions in molecular docking. A more negative binding energy value indicates stronger receptor-ligand affinity (Paggi et al., 2024). Figure 6 shows the 3D and 2D interaction diagrams of representative complexes.

Using molecular docking, we evaluated the interactions of four bioactive compounds from AM with eight critical target genes. The threshold for good binding activity is generally a binding energy less than −5.0 kcal·mol^−1^. Binding energies exceeding −7.0 kcal·mol^−1^ (i.e., more negative) represent superior binding affinity and more substantial interactions (Hui et al., 2021). The docking results consistently demonstrated favorable binding activity, pointing to the presence of interactions between the active components of AM and the eight key targets at a molecular level. (For molecular docking lattice parameters, see Supplementary Appendix Table A5).

Table 2 indicates that the four active components of AM interacted with eight key target genes, exhibiting binding affinities ranging from −10.6 kcal·mol^−1^ to −6.4 kcal·mol^−1^, where lower docking scores signify stronger binding affinity. The docking results consistently demonstrated favorable binding activity, pointing to the presence of interactions between the active components of AM and the eight key targets at a molecular level. (For molecular docking lattice parameters, see Supplementary Appendix Table A5).

**TABLE 2:** Binding energies between selected active components of AM and key target proteins.

Core target	Compound	PDB-ID	Binding affinity
IL-6	AS-Ⅳ	1ALU	−10.6 kcal·mol^-1^
TNF-α	AS-Ⅳ	1A8M	−7.9 kcal·mol^-1^
IL-1β	AS-Ⅳ	1HIB	−7.5 kcal·mol^-1^
IL-10	AS-Ⅳ	1ILK	−8.4 kcal·mol^-1^
ALB	AS-Ⅳ	6JE7	−9.0 kcal·mol^-1^
IL-4	calycosin	1BBN	−6.4 kcal·mol^-1^
MMP-9	kaempferol	1ITV	−8.4 kcal·mol^-1^
TGF-β1	formononetin	8UDZ	−8.6 kcal·mol^-1^

Network pharmacology and molecular docking analysis suggest that the active components of AM may intervene in AD through multiple pathways, involving inflammation suppression, immune regulation, epidermal barrier repair, and regulation of the GSA homeostasis. KEGG enrichment analysis indicates that the NF-κB signaling pathway may be a key pathway for AM intervention in AD. This pathway is known to regulate the expression of pro-inflammatory factors such as IL-6, TNF-α, and IL-1β. Molecular docking results further revealed that AS-IV exhibits high binding affinity with targets including IL-6 and TNF-α, suggesting its potential role in regulating inflammatory responses by acting on these targets. These computational simulations provide preliminary mechanistic evidence for AM’s regulation of inflammation and immune homeostasis, consistent with its traditional applications and existing experimental reports.

### 6.7 Molecular dynamics simulation

Protein folding, ligand interactions, and PPI are complex processes that defy full elucidation through experimental observation alone. The investigation of protein motion in molecular dynamics simulations relies on tracking conformational changes as a function of time (Collier et al., 2020). This technique is ubiquitously employed to evaluate the binding stability and conformational adaptability of active compounds with therapeutic targets (Liu Y. et al., 2023). Molecular dynamics simulations were conducted using Gromacs (Version 2023.2) (Pronk et al., 2013) to assess the binding stability of the AS-IV/IL-6 and AS-IV/TNF protein-ligand complexes. The protein-ligand complex was modeled using the Amber99sb-ild force field, with hydrogenation and RESP (restrained electrostatic potential) calculations performed via Gaussian16W. Solvation of the system was performed using a dodecahedral box of TIP3P water, and Na^+^ was added at a concentration of 0.154 mol·L^−1^ to neutralize the system’s charge. In molecular dynamics simulations of this system (300 K, 1 bar), initial equilibration was performed under NVT conditions for 100 ps at constant temperature, volume, and pressure. Throughout the equilibration phase, constrained atoms were fixed at their initial coordinate positions. Following equilibration, a production simulation was conducted under identical temperature and pressure conditions for 100 ns with a time step of 2 fs. Gromacs’ built-in tools were used to calculate the ligand’s root mean square deviation (RMSD), root mean square fluctuation (RMSF), radius of gyration (RG), solvent accessible surface area (SASA), and hydrogen bond count (Fen et al., 2025; Lu et al., 2023).

**FIGURE 7:**
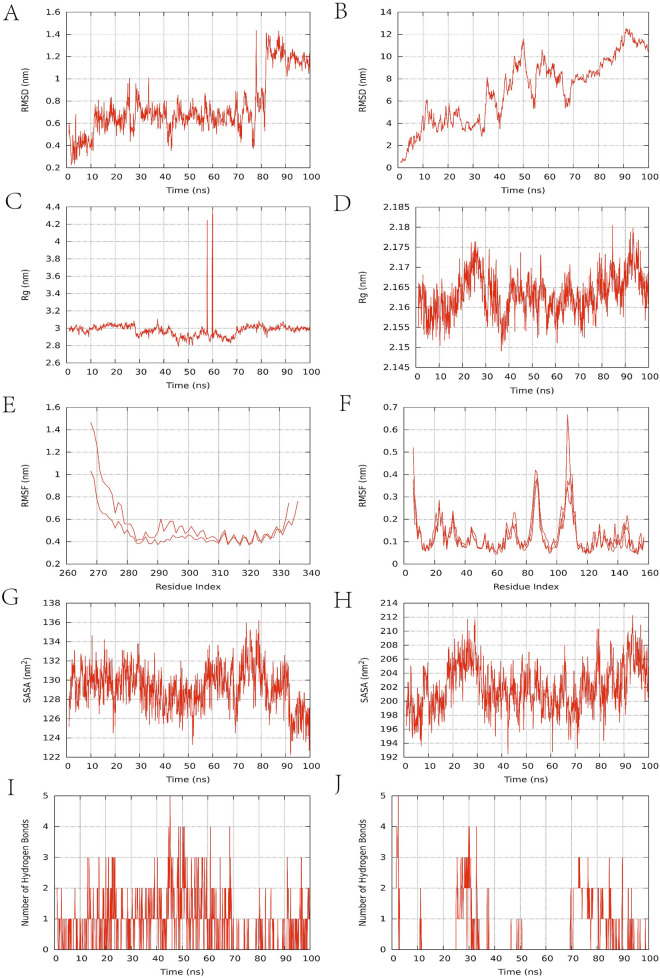
Molecular dynamics simulation results. **(A,B)** RMSD change curve; **(C,D)** Rg curve; **(E,F)** amino acid residue fluctuation; **(G,H)** SASA fluctuation curve; **(I,J)** number of hydrogen bonds. The left side of Figure 7 represents the IL-6 complex, and the right side represents the TNF-α complex.

RMSD trajectories are routinely analyzed to evaluate the structural persistence of the protein-ligand complex, with lower values indicating more stable binding. The AS-IV/IL6 complex (Figure 7A) exhibits minimal RMSD fluctuations between 10 and 80 ns (0.4–1.0 nm), indicating overall stability. The TNF-α complex (Figure 7B) fluctuates within a range of 0–14 nm, indicating that the system has not yet reached a steady state. Rg is typically used to assess the folding or unfolding of protein-ligand complexes, providing insights into the compactness of the complex. The Rg value of the AS-IV/IL6 complex (Figure 7C) is around 3.0 nm, with significant fluctuations only observed between 58 and 60 ns, indicating that the protein is folded stably; the Rg value of the TNF complex (Figure 7D) remains stable around 2.16 nm. RMSF reflects the fluctuations of protein amino acid residues. The IL-6 complex (Figure 7E) exhibits significant residue fluctuations (up to 1.4 nm), while the TNF-α complex (Figure 7F) shows minimal fluctuations (<0.6 nm). SASA can be used to characterize changes in protein hydrophilicity, with lower values indicating that the protein molecules are more tightly packed. The SASA of the IL6 complex (Figure 7G) remained stable around 130 nm^2^, while the SASA of the TNF complex (Figure 7H) was higher (approximately 200 nm^2^), indicating that the protein in the IL-6 complex wraps more tightly around the ligand. Among non-covalent interactions, hydrogen bonding ranks as one of the strongest, and the number of hydrogen bonds observed in simulations correlates with binding strength. The IL6 complex (Figure 7I) formed 3-5 hydrogen bonds, with relatively frequent hydrogen bond interactions contributing to binding. In terms of the persistence of hydrogen bond formation, the stability of the TNF-α complex is weaker than that of the IL-6 complex (Figure 7J). Overall, the AS-IV/IL-6 and AS-IV/TNF-α complex systems exhibit significant differences in dynamic stability, with preliminary results indicating superior stability for the former complex.

### 6.8 The role of key targets in gut health

Based on computer-based prediction and simulation, we preliminarily identified IL-6, TNF-α, and NF-κB as potential key targets for AM intervention in AD. Although this study did not directly conduct functional experiments to validate the specific roles of these targets within the GSA, existing literature suggests that they also play crucial roles in maintaining intestinal immune regulation and barrier function. This provides a theoretical bridge for understanding how AM exerts its systemic regulatory effects through the GSA.

IL-6, TNF-α, and NF-κB are not only core regulators of skin inflammation in AD but also extensively involved in intestinal mucosal immunity and barrier stability. IL-6 is a key cytokine that promotes intestinal inflammation, with elevated levels closely correlated with inflammatory severity. Experimental studies indicate that certain commensal *E. coli* strains (such as ST129 and ST375) exacerbate DSS-induced intestinal inflammation by inducing massive IL-6 production in the host, while blocking IL-6 significantly mitigates pathological damage (Kittana et al., 2018). Research indicates that IL-6 plays a dual role in intestinal inflammation: on one hand, it exacerbates inflammation by promoting immune cell activation and the release of inflammatory mediators; on the other hand, it also participates in mucosal repair and maintains intestinal barrier function (Guo et al., 2021). TNF-α also exhibits a dual role in intestinal homeostasis: under physiological conditions, it promotes intestinal barrier repair and immune regulation, while excessive activation in pathological states drives inflammation, cell death, and tumorigenesis (Ruder et al., 2019). In a rat model of intestinal inflammation, neutralizing IL-6 and TNF-α significantly improved intestinal permeability and mucosal damage. The mechanisms involved were associated with the inhibition of claudin-2 and MLCK expression, respectively, suggesting that jointly targeting these two cytokines may provide more effective protection for the intestinal barrier (Xiao et al., 2016). As a central transcription factor hub regulating intestinal immune homeostasis and inflammation, NF-κB’s moderate activation supports intestinal barrier integrity through the induction of key mediators, including proinflammatory cytokines, chemokines, and antimicrobial peptides. However, sustained NF-κB signaling disrupts the expression and distribution of tight junction proteins (such as ZO-1 and occludin), leading to increased intestinal permeability, accelerated epithelial cell apoptosis, and impaired mucosal repair, thereby driving the onset and progression of inflammatory bowel disease (Mukherjee et al., 2024). Additionally, NF-κB participates in regulating inflammatory responses through both classical and non-classical signaling pathways, while simultaneously influencing T cell differentiation, IgA secretion, and intestinal barrier integrity, playing a crucial role in maintaining intestinal immune homeostasis (Wang et al., 2022c). In summary, this study suggests that the potential target molecules for AM intervention in AD may be IL-6, TNF-α, and NF-κB. Existing literature further supports that AM may reproduce its known protective mechanism in the gut by synergistically regulating these target molecules—namely, enhancing intestinal barrier function through modulating tight junction protein expression via the claudin-2/MLCK pathway. This discovery provides clear molecular pathway evidence supporting the hypothesis that AM mitigates AD through the GSA, expanding its mechanism of action from local skin anti-inflammation to systemic regulation of intestinal immunity and barrier function.

## 7 Safety evaluation of AM

AM has demonstrated high safety in clinical preparations, extracts, and single compounds, with low acute and long-term toxicity and low risk associated with short-term high-dose use (Zhou et al., 2018). AM injection demonstrated low acute toxicity in mouse and rat models (intravenous LD_50_ = 90.39 g/kg, intraperitoneal LD_50_ = 108.11 g/kg), far exceeding clinical doses. Furthermore, no significant long-term toxicity was induced by high-dose intraperitoneal administration for 30 consecutive days (Han et al., 2004). Shenqi Fuzheng Injection is a prescription drug approved for market release, with its primary ingredients being AM and Codonopsis pilosula. Long-term toxicity studies indicate no chronic toxic reactions were observed in rabbits after 90 days of continuous administration. Post-marketing safety surveillance data reported 23 adverse reactions, primarily including thrombocytopenia, palpitations, and eyelid edema. This indicates potential toxicity associated with AM and necessitates a comprehensive assessment of long-term safety (Ai et al., 2014). Note that AM may possess potential toxicity; the safety of long-term use should be comprehensively evaluated. Safety studies in rabbits confirmed that AM freeze-dried powder for injection caused no hemolysis, irritation, or allergic reactions. Its acute toxicity is low, with maximum tolerated doses for intravenous and intraperitoneal administration (200 and 400 g/kg) being 350 times and 700 times the clinical daily dose (0.57 g/kg), respectively. This indicates the drug possesses high clinical safety (Yang et al., 2007). Animal studies showed that oral administration of AM decoction (45–180 g/kg) for 90 consecutive days produced no significant effects on rat body weight, hematological and urinary parameters, major organ coefficients, or hepatic and renal function. These findings collectively indicate a favorable safety profile for AM decoction, supporting its long-term use at relatively high doses (Liu et al., 2009). According to reports, the maximum tolerated dose (MTD) of APS for acute toxicity is > 15 g/kg bw. Results from the Ames test, bone marrow micronucleus test, sperm deformity test, and 30-day feeding test were all negative, confirming that it has no genetic toxicity and no obvious subchronic toxicity (Zhao et al., 2009). At 1.0 mg/kg, AS-IV manifested maternal toxicity (reduced gestational weight gain) and embryotoxicity (decreased fetal viability with elevated fetal death) in Sprague-Dawley rats, but induced no maternal toxicity in rabbits. No teratogenic effects were observed in either species, with the highest tested doses (1.0 mg/kg in rats, 2.0 mg/kg in rabbits) yielding no significant external, visceral, or skeletal malformations in fetuses (Zhu et al., 2007; Zhu et al., 2010). Two completed Phase I clinical trials have shown that AS-IV Glucose Injection (100 mL once daily) and Total Astragalosides Injection (100–200 mg/mL once daily) were well tolerated in healthy subjects, with only isolated cases of mild adverse events (e.g., phlebitis). These findings established safe dosage ranges for Phase II clinical trials (Chen et al., 2013; Cheng et al., 2012). Research indicates that formononetin, a phytoestrogen derived from AM, exhibits significant acute toxicity in mice (LD_50_ = 103.6 mg/kg), with lethal liver damage occurring at a dose of 300 mg/kg. Its subacute toxicity No Observed Adverse Effect Level (NOAEL) was determined to be 50 mg/kg (Tay et al., 2019). Similarly, the proper use of AM preparations is safe and well-tolerated. Song et al. demonstrated that the AM-containing herbal formula HT042 (composed of AM, *Eleutherococcus senticosus*, and *Phlomis umbrosa*) and its constituent botanical drugs exhibit low acute oral toxicity (LD_50_ > 5000 mg/kg) in rats, with a no-observed-adverse-effect level of 4,000 mg/kg/day established for the HT042 formula in a 13-week subchronic toxicity study (Song et al., 2017).

Additionally, pharmacokinetic studies indicate that APS exhibits rapid absorption following intravenous administration (T_max_ = 0.67 ± 0.26 h) but slow elimination (T_1_/_2_β = 0.44 ± 0.05 h, MRT0- ∞ = 34.38 ± 12.59 h), primarily metabolized via non-renal pathways. The pharmacokinetic curve demonstrates prolonged retention time in the body and the presence of enterohepatic circulation (Wang T. et al., 2025). In summary, existing evidence indicates that AM and its primary active components demonstrate high preclinical and clinical safety at both conventional and higher doses, with a broad therapeutic window and low risk associated with long-term use. However, specific constituents such as AS-IV and formononetin may exhibit potential toxicity at elevated doses, suggesting that safety evaluations for long-term administration remain warranted.

## 8 Adverse drug interactions and implications for future therapeutic applications

Drug-drug interactions are a critical factor affecting clinical treatment safety, particularly serving as an important warning for disease management requiring long-term combination therapy. In recent years, AM and its active components (such as AS-IV, APS, and flavonoids) have demonstrated multiple potentials in AD treatment. However, the risk of interactions when combined with conventional Western medications has not been systematically evaluated. Therefore, investigating whether AM components interfere with the activity of drug transporters (e.g., P-gp) and metabolic enzymes (e.g., CYP450) is crucial for ensuring the safety of combination therapy and advancing its clinical translation. Studies indicate that AM and its components (AS-IV, calycosin, formononetin) can induce the expression and activity of P-gp and breast cancer resistance protein via the Nrf2 pathway, suggesting potential drug interactions when co-administered with relevant substrate drugs (Lou et al., 2019). The CYP450 enzyme family plays a pivotal role in drug metabolism, with CYP3A4 being a key isoform within this family. Research has confirmed that AM is an inducer of CYP3A4. By upregulating CYP3A4 expression, it accelerates the metabolism of substrates such as tacrolimus, leading to a significant reduction in the blood concentrations of these drugs (Bukowska et al., 2025; Lau et al., 2013), increasing the risk of toxicity. Tacrolimus, a commonly used calcineurin inhibitor for treating AD, reduces inflammatory responses by suppressing T-cell activation. Its combination with astragalus may carry the risk of diminished efficacy or even treatment failure (Zhang et al., 2025d), warranting clinical attention. AM water extract exhibits dose-dependent induction of protein and gene expression for multiple CYP450 enzyme subtypes (including CYP3A4, CYP2B6, and CYP2E1), suggesting potential pharmacokinetic interactions when combined with drugs metabolized via CYP450 pathways (Zhang et al., 2016). Recent studies indicate that AM injection has demonstrated inhibitory effects on multiple CYP450 enzyme activities in the latest research, while simultaneously enhancing the activity of certain subtypes. This further suggests its potential to regulate the CYP450 system, a key liver drug metabolism enzyme (Shi et al., 2025). Other studies have shown that AM and its active components exert complex bidirectional regulatory effects on the CYP450 enzyme system. These effects include concentration-dependent inhibition (e.g., of CYP2C9 and CYP2D6) as well as induction of expression in multiple subtypes (e.g., CYP3A4 and CYP2E1), demonstrating both component-specificity and enzyme subtype-specificity (Wang X. et al., 2025). In animal studies, AM injection inhibited the enzymatic activity of CYP2D6, CYP2C19, and CYP1A2 in rats *in vitro*, while *in vivo* it induced CYP1A2 and CYP3A1 activity (Zhang et al., 2013a; Zhang et al., 2013b). Although AM possesses therapeutic potential, its constituents can complexly regulate drug-metabolizing enzymes and transporters. Concurrent use with Western medications (such as tacrolimus) may diminish therapeutic efficacy, necessitating caution in clinical combination therapy.

## 9 Result

The traditional efficacy of AM in treating AD aligns closely with the principles of pattern differentiation and treatment in TCM, particularly benefiting patients in remission with lung-spleen qi deficiency and compromised defensive-surface integrity. Clinically, AM alleviates AD symptoms by fortifying qi and consolidating the surface, strengthening the spleen, and dispelling dampness. It is often combined with blood-nourishing botanical drugs in compound formulas to enhance qi, generate blood, moisten dryness, and relieve itching. At the pharmacological level, AM and its active components (such as polysaccharides, saponins, and flavonoids) exhibit multiple actions related to core AD pathogenesis factors. These include regulating Th17/Treg immune balance, suppressing pro-inflammatory factor expression (e.g., IL-6, TNF-α), repairing skin barrier function, and counteracting oxidative stress. These actions directly intervene in AD’s immune dysregulation and inflammatory cascade. Further studies reveal that AM reshapes gut microbiota composition, promotes SCFA production, enhances tight junction protein expression, and improves intestinal barrier function. This provides substantial evidence for its regulation of systemic inflammation and skin immunity via the GSA, suggesting AM may inhibit inflammatory signal migration to the skin through gut-derived mechanisms. Network pharmacology enrichment analysis identified NF-κB and IL-17 signaling pathways as key regulatory hubs. Molecular docking results demonstrated a strong binding affinity (binding energy reaching −10.6 kcal/mol^−^) between components, such as AS-IV, and core targets, including IL-6 and TNF-α. Molecular dynamics simulations further validated the dynamic stability of the AS-IV/IL-6 complex. Moreover, these targets and pathways also serve as key regulators of intestinal immune modulation and barrier function. This further reinforces, at the molecular level, the pathophysiological basis for AM intervention in AD via the GSA, while also providing new directions for future in-depth research in this area.

## 10 Discussion

This study systematically elucidates the multidimensional mechanisms of action of AM and its active components in treating AD by integrating traditional application experience with modern pharmacological evidence. Unlike previous research focusing on single components or localized skin effects, the core advancement of this work lies in introducing a GSA regulation perspective to explain the key role of AM in intervening AD. AM and its active components can effectively inhibit the migration of intestinal inflammatory factors to the skin by reshaping the gut microbiota structure, promoting the production of SCFAs (such as butyrate), and enhancing intestinal barrier function. This finding aligns with TCM theories that “the lungs govern the skin and hair” and “The lung and large intestine are interiorly-exteriorly related.” It provides a modern biological interpretation for the holistic concept of “treating skin diseases by addressing the gut” from the perspective of gut microbiota. It provides preliminary evidence supporting the efficacy and potential of AM in treating AD. Network pharmacology and computational modeling results collectively suggest that AM exerts synergistic therapeutic effects by intervening in key inflammatory pathways such as IL-17 and NF-κB, highlighting its holistic regulatory advantage of “multicomponent-multitarget-multipathway” action. The study preliminarily validated the high affinity and binding stability between core components like AS-IV and key targets such as IL-6 and TNF-α. These dynamic details, difficult to capture through traditional experiments, offer new insights and hypotheses for understanding the precise molecular basis of AM’s pharmacological effects, while also providing clues for subsequent experimental validation and drug optimization.

However, this study still has several limitations. First, existing evidence primarily comes from animal experiments and *in vitro* studies, lacking large-scale randomized controlled clinical trial data in AD patients. The efficacy, optimal dosage, and long-term safety of AM in real-world settings remain to be determined. Second, although network pharmacology analysis suggests synergistic potential among AM’s active components, the specific mechanisms of these synergistic effects require experimental validation. We propose an integrated mechanism for AM modulating AD via GSA, supported by multi-omics data and computational simulations. However, this pathway has not yet undergone causal validation through functional experiments (e.g., microbiota transplantation, metabolite intervention). Furthermore, while molecular docking and kinetic simulations indicate binding potential, the biological functionality requires further confirmation via surface plasmon resonance and reporter gene assays.

Looking ahead, priority should be given to conducting high-quality clinical studies using standardized outcome measures such as SCORAD and EASI to validate the efficacy and safety of AM monotherapy or adjunctive therapy in AD patients. To elucidate the “multi-component mechanism” of AM, future studies should evaluate the anti-inflammatory and barrier-repairing effects of APS, saponins, and flavonoids—both individually and in combination—in AD animal models. Utilizing isoradiation plots to calculate the synergy index (CI) will quantify interactions among these components. Further integration of transcriptomic and proteomic analyses will provide empirical evidence for AM’s multi-target action mechanisms. At the mechanistic level, gut microbiota transplantation and metabolomics analysis should be employed to elucidate the specific pathways through which AM modulates GSA and its causal role in AD pathogenesis. Additionally, a systematic assessment of pharmacokinetic interactions between AM and biological agents, immunosuppressants, and other medications should be conducted to provide a basis for developing safe combination therapy regimens in clinical practice.

In summary, this study provides preliminary pharmacological rationale and mechanistic insights for AM treatment in AD, demonstrating promising prevention and therapeutic potential. However, current research remains limited, particularly in clinical translation, facing challenges such as insufficient human data, unclear mechanisms of action, and undefined drug interactions. Future efforts should focus on clinical needs, deepen mechanism research, and promote the scientific, safe, and effective application of AM in the comprehensive treatment of AD.
