# Supplementary material for: The therapeutic potential of Astragalus membranaceus in atopic dermatitis: from traditional applications and modern pharmacological research to regulation of the Gut-Skin Axis
Source: Front Pharmacol. 2025 Nov 7;16:1685708. doi: 10.3389/fphar.2025.1685708 (PMC12634398; doi:10.3389/fphar.2025.1685708)
Supplement: Supplementary file 1 [file Table1.docx]

**Table.A1 The composition of all complex preparations - Yupingfeng Powder**

| **Botanical Drug (Latin Name)** | **Used in Extract (Ratio)** | **Converted Dosage (g)** |
| --- | --- | --- |
| *Astragalus membranaceus* Fisch. ex Bunge | **3** | **90g** |
| *Atractylodes macrocephala* Koidz. | **1** | **30g** |
| *Saposhnikovia divaricata* (Turcz. ex Ledeb.) Schischk. | **1** | **30g** |

**Table.A2 The composition of all complex preparations - Danggui Buxue Tang**

| **Botanical Drug**  **(Latin Name)** | **Used in Extract (Ratio)** | **Converted Dosage (g)** |
| --- | --- | --- |
| *Astragalus membranaceus* Fisch. ex Bunge | **5** | **30g** |
| *Angelica L.* | **1** | **5g** |

**Table.A3 The composition of all complex preparations - Spleen-Nourishing, Blood-Nourishing, Wind-Dispelling Decoction**

| **Botanical Drug**  **(Latin Name)** | **Used in Extract (Ratio)** | **Converted Dosage (g)** |
| --- | --- | --- |
| *Astragalus membranaceus* Fisch. ex Bunge | 6 | 30g |
| *Atractylodes macrocephala* Koidz. | 3 | 15g |
| *Angelica L.* | 3 | 15g |
| *Saposhnikovia divaricata* (Turcz. ex Ledeb.) Schischk. | 4 | 20g |
| *Tribulus terrestris Muhl.* | 6 | 30g |
| *Centipede* | 1 | 5g |

**Table. A4 Active compounds of AM derived from the TCMSP database**

| **MOL_ID** | **Molecule** | **OB（%）** | **DL** |
| --- | --- | --- | --- |
| MOL000211 | Mairin | 55.37 | 0.77 |
| MOL000239 | Jaranol | 50.82 | 0.29 |
| MOL000296 | hederagenin | 36.91 | 0.75 |
| MOL000033 | (3S,8S,9S,10R,13R,14S,17R)-10,13-dimethyl-17-[(2R,5S)-5-propan-2-yloctan-2-yl]-2,3,4,7,8,9,11,12,14,15,16,17-dodecahydro-1H-cyclopenta[a]phenanthren-3-ol | 36.22 | 0.78 |
| MOL000354 | isorhamnetin | 49.60 | 0.30 |
| MOL000371 | 3,9-di-O-methylnissolin | 53.74 | 0.47 |
| MOL000378 | 7-O-methylisomucronulatol | 74.68 | 0.29 |
| MOL000379 | 9,10-dimethoxypterocarpan-3-O-β-D-glucoside | 36.73 | 0.92 |
| MOL000380 | (6aR,11aR)-9,10-dimethoxy-6a,11a-dihydro-6H-benzofurano[3,2-c]chromen-3-ol | 64.25 | 0.42 |
| MOL000387 | Bifendate | 31.09 | 0.66 |
| MOL000392 | formononetin | 69.67 | 0.21 |
| MOL000403 | astragalosideII | 46.05 | 0.12 |
| MOL000407 | astragalosideⅣ | 22.50 | 0.15 |
| MOL000417 | Calycosin | 47.75 | 0.24 |
| MOL000422 | kaempferol | 41.88 | 0.24 |
| MOL000432 | linolenicacid | 45.00 | 0.14 |
| MOL000433 | FA | 68.96 | 0.70 |
| MOL000435 | acetylastragalosideI_qt | 30.75 | 0.17 |
| MOL000438 | (3R)-3-(2-hydroxy-3,4-dimethoxyphenyl)chroman-7-ol | 67.66 | 0.26 |
| MOL000439 | isomucronulatol-7,2'-di-O-glucosiole | 49.28 | 0.62 |
| MOL000098 | quercetin | 46.43 | 0.27 |

**Table.A5 Center and Size of the Docking Lattice for Protein Targets**

| **protein target** | **PDB ID** | **Lattice center X (Å)** | **Lattice center Y (Å)** | **Lattice center Z (Å)** | **Lattice size X (Å)** | **Lattice size Y (Å)** | **Lattice size Z (Å)** |
| --- | --- | --- | --- | --- | --- | --- | --- |
| IL-6 | 1ALU | **29.605** | **56.518** | **32.899** | **62** | **116** | **50** |
| TNF-α | 1A8M | **19.943** | **48.943** | **42.163** | **102** | **106** | **96** |
| IL-1B | 1HIB | **23.507** | **2.071** | **75.049** | **102** | **98** | **88** |
| IL-10 | 1ILK | **24.947** | **43.937** | **39.708** | **102** | **106** | **70** |
| ALB | 6JE7 | **0.206** | **-23.127** | **28.613** | **86** | **92** | **114** |
| IL-4 | 1BBN | **-0.018** | **1.291** | **2.073** | **126** | **96** | **68** |
| MMP-9 | 1ITV | **54.92** | **21.248** | **109.498** | **86** | **74** | **126** |
